# Advancing Depression Management Through Biomarker Discovery with a Focus on Genetic and Epigenetic Aspects: A Comprehensive Study on Neurobiological, Neuroendocrine, Metabolic, and Inflammatory Pathways

**DOI:** 10.3390/genes16050487

**Published:** 2025-04-25

**Authors:** Jelena Milic, Sladjana Jovic, Rosa Sapic

**Affiliations:** 1 Institute of Public Health of Serbia “Dr Milan Jovanovic Batut”, Dr. Subotica Starijeg 6, 11000 Belgrade, Serbia; 2 Faculty of Nursing, Serbia European University KALLOS, Gospodara Vucica 40, 11000 Belgrade, Serbia; 3 Faculty of Security Studies, University of Belgrade, Gospodara Vucica 40, 11000 Belgrade, Serbia; sladjana.jovic@fb.bg.ac.rs; 4 Faculty of Health Studies, University of Bijeljina, 76300 Bijeljina, Republika Srpska, Bosnia and Herzegovina; sapicdr@gmail.com

**Keywords:** depression, genetic biomarkers, epigenetic biomarkers, biomarkers, diagnostic tools, personalized therapies, treatment outcomes, neurobiological pathways

## Abstract

**Introduction:** Depression is a pervasive global health issue, affecting millions worldwide and causing significant disability. Despite its prevalence, current diagnostic and treatment approaches often yield suboptimal outcomes. The complexity of depression, characterized by diverse causes and symptoms, highlights the urgent need for advanced diagnostic tools and personalized therapies. Biomarkers, particularly **genetic** and **epigenetic** depression biomarkers, offer promise in uncovering the biological mechanisms underlying depression, potentially revolutionizing its management. **Aim: Primary aim:** To identify biomarkers associated with depressive disorders, with a focus on **genetic** and **epigenetic biomarkers**. **Secondary aim:** To optimize the current classification of biomarkers associated with different types of depressive disorders, with a focus on **genetic** and **epigenetic biomarkers**. **Methods:** We integrated findings with strategic keywords extracted from relevant studies, conducting a thorough literature review across the Google Scholar, PubMed, and Web of Science databases. Lastly, final reference inclusion had stringent criteria: recent, diverse peer-reviewed articles in English, all study designs, ensuring up-to-date coverage of **genetic** and **epigenetic depression biomarker** research. **Results:** The review reveals the classification of **genetic** and **epigenetic biomarkers** in regard to the type of biomarker, the system of the human body it derives from, and the sampling entity. All of the findings show promise in diagnosing depression, with the potential of predicting treatment outcomes and guiding personalized therapeutic approaches. We defined the significant correlations between **genetic** and **epigenetic biomarker** profiles and clinical parameters such as symptom severity and treatment response, thereby enhancing diagnostic accuracy and guiding treatment strategies tailored to individual patient needs across diverse depressive subtypes and treatment responses. **Conclusion:** Identifying biomarkers associated with depressive disorders, with a focus on **genetic** and **epigenetic biomarkers**, represents a critical step toward improving diagnostic precision and treatment efficacy. By elucidating the complex biological underpinnings of depression, this study contributes to the development of targeted therapies that address the diverse needs of individuals affected by this debilitating group of disorders. Future research should focus on validating these **genetic** and **epigenetic biomarkers** in larger cohorts and clinical trials to facilitate their clinical implementation and enhance patient outcomes.

## 1. Introduction

Here is a light-hearted take on the importance of identifying depression: Why did the doctor insist on identifying depression in his patients? Because it is important to address their “down payments” before they accrue interest. The complexity of manifestation of depression—often intertwined with comorbid conditions such as anxiety or circadian rhythm changes (sleep and eating disorders)—further underscores the urgent need for precise biomarkers to aid early diagnosis and treatment [1]. On a more serious note, if the intensity of human suffering were the sole criterion for prioritizing medical conditions, depression would demand urgent attention. Affecting over 350 million people globally, it remains the leading cause of health-related burden worldwide [2], exerting a profound impact both personally and societally [3]. Its consequences extend far beyond mental health, impairing daily functioning and contributing to a substantial share of suicides—approximately two-thirds of suicide victims suffer from depression. Epidemiological data indicate that 59–87% of suicide victims had major depression, and up to 15% of these patients eventually die by suicide [4].

Focusing specifically on major depressive disorder (MDD), its prevalence continues to rise at an alarming rate. Between 2005 and 2018, the number of U.S. adults diagnosed with MDD increased from 13.7 million to 17.5 million, with the prevalence rate climbing from 6.8% to 7.1% [5,6]. This growth is particularly pronounced in individuals aged 18–34 [6]. The COVID-19 pandemic further exacerbated this trend. Beginning in early 2020, factors such as health-related fears, job insecurity, and prolonged social isolation led to heightened psychological distress. Data from the Centers for Disease Control and Prevention (CDC) show that the prevalence of MDD rose from 7% pre-pandemic to 27% during the pandemic’s first year. Co-occurring anxiety disorders also increased, from 11% to 38% [7,8].

This prevalent mental disorder often presents with a wide range of debilitating symptoms, including severe functional impairment and, in extreme cases, suicidal ideation. Despite its growing impact, the clinical diagnosis of depression still relies heavily on subjective methods such as psychological evaluations and structured interviews, which can sometimes result in misdiagnosis and ineffective treatment strategies. As such, the identification of high-quality, biologically grounded biomarkers and the development of reliable, accessible tools for early diagnosis have become critical objectives. This review provides a comprehensive overview of emerging biomarkers for depression, with the goal of enhancing diagnostic accuracy and guiding personalized treatment approaches [9].

Despite projections from the World Health Organization that depression will be the leading global cause of disease burden by 2030 [10], it remains significantly underdiagnosed and undertreated. In the U.S., an estimated two-thirds of depression cases go undiagnosed [11]. Existing treatments yield full remission in only 28% of cases, with reduced effectiveness upon repeated interventions [12]. Misconceptions such as the simplistic “chemical imbalance” theory [13] have historically limited treatment approaches, while stigma continues to isolate patients and deter them from seeking help [14].

Beyond individual suffering, depression imposes enormous economic costs. Workplace-related expenses form the largest share of MDD’s growing economic burden, particularly among working-age adults. While the number of diagnosed cases has risen, per-person treatment costs have decreased, highlighting increased access without necessarily improved outcomes. Despite a growing number of individuals receiving care, significant gaps in treatment persist. This underscores the urgent need for rapid, reliable diagnostic tools and more effective interventions. At the forefront of these advancements is the exploration of genetic and epigenetic biomarkers—biological indicators that offer potential for unraveling the underlying mechanisms of depression and transforming its clinical management [6].

### 1.1. Biomarkers in Depression Research

Biomarkers are a rapidly expanding area in psychiatric research, providing critical insights into the diverse biological mechanisms underpinning depression [15]. These markers span several systems—neurobiological, neuroendocrine, metabolic, and inflammatory—all implicated in the disorder’s pathophysiology [15]. Previously published evidence emphasizes the need for sophisticated, integrative research to understand inflammation’s role in MDD [16]. Progress in neuroimaging, genetic profiling, and biochemical analysis has deepened our understanding of depression’s molecular basis [15,17,18,19,20,21], revealing complex interactions between neurotransmitter dysfunction, neuroendocrine imbalance, and immune activation [22].

Specific genetic and epigenetic biomarkers have been associated with depression severity, therapeutic responsiveness, and prognosis [15,23]. For instance, alterations in neurotrophic factors, such as brain-derived neurotrophic factor (BDNF) [24] and inflammatory cytokines [25], have been consistently linked to depressive symptoms. These biomarkers not only aid diagnosis but also open avenues for personalized medicine, enabling tailored treatment plans that optimize efficacy and reduce adverse effects.

Integrating these biomarkers into clinical practice could revolutionize depression care by supporting early detection, guiding therapy selection, and monitoring progression. However, translating these discoveries into practice requires extensive validation via large-scale, longitudinal studies to ensure reliability and reproducibility across diverse populations.

### 1.2. Identifying and Validating Biomarkers Associated with Depression

Depression remains a major and escalating public health challenge, yet its diagnosis relies solely on clinical assessment, with no established biological markers in routine use. Biomarkers—objectively measurable indicators of physiological or pathological processes—could provide much-needed clarity in identifying depression earlier and predicting treatment response, particularly in cases of medication resistance [26]. Identifying and validating genetic and epigenetic biomarkers represents a pivotal step in enhancing depression diagnosis and treatment. By decoding the complex biological underpinnings of this pervasive disorder, researchers aim to develop more accurate diagnostics and targeted therapies that accommodate individual variability in treatment response.

Molecular biomarkers are emerging as valuable tools for advancing clinical psychiatry, offering potential to enhance diagnostic accuracy and treatment personalization. In this context, major depressive disorder (MDD) has become a focal point of biomarker research, not only due to its rising global prevalence but also its complex, multifactorial pathophysiology. Major depressive disorder is a biologically heterogeneous condition lacking reliable biomarkers to assess severity, subtypes, or treatment response. A growing body of evidence indicates that alterations in peripheral growth factors, cytokines, endocrine, and metabolic markers are implicated in MDD pathophysiology and may inform treatment outcomes. These findings underscore the urgent need for the development of an integrated biomarker panel that captures the biological diversity of MDD, enabling more precise diagnosis and individualized therapeutic strategies [27].

### 1.3. Aim

Primary aim: To identify biomarkers associated with depressive disorders, with a focus on genetic and epigenetic biomarkers.

Secondary aim: To optimize the current classification of biomarkers associated with different types of depressive disorders, with a focus on genetic and epigenetic biomarkers.

## 2. Methods

In our research methodology, we strategically selected additional terms extracted from abstracts of pertinent studies. This approach was pivotal in refining our search syntax to compile a comprehensive reference list for our review article. During this phase, particularly effective when finding relevant studies, we used keywords such as “markers of depression”, “biomarkers”, and “diagnostic tools”. Our systematic literature search was conducted across reputable databases including Google Scholar, PubMed, and Web of Science. Integrating these keywords into our search strategy based on abstracts of relevant studies ensured a thorough synthesis of current knowledge on genetic and epigenetic depression biomarkers, among other depression-related biomarkers. This synthesis was instrumental in informing the development of a robust reference list that formed the scientific foundation of our review article.

To guide and organize our literature search, we employed the PICO framework. The PICO process (or framework) is a mnemonic used in evidence-based practice, specifically in evidence-based medicine, to frame and answer clinical or healthcare-related questions. It is widely used to develop literature search strategies, particularly in systematic reviews. The PICO acronym stands for:

P—Patient, problem, or population;
I—Investigated condition (e.g., intervention, exposure, risk/prognostic factor, or test result);
C—Comparison condition (e.g., intervention, exposure, risk/prognostic factor, or test result, respectively);
O—Outcome(s) (e.g., symptom, syndrome, or disease of interest).

In our study, we used the PICO framework to refine the search strategy for biomarkers of depression, with a focus on genetic and epigenetic depression biomarkers among those identified. Specifically, the patient population was individuals with depressive disorders, the investigated condition was biomarkers of depression (with a particular focus on genetic and epigenetic depression biomarkers), the comparison condition involved existing biomarkers or diagnostic tools, and the outcome focused on the diagnostic accuracy, severity correlation, and predictive potential of the biomarkers.

The criteria for final inclusion in our reference list were stringent, focusing on recent peer-reviewed publications that employed advanced biochemical methodologies, particularly those related to genetic and epigenetic depression biomarkers. Articles had to be published in English and encompass diverse study designs, reflecting the dynamic and evolving nature of research in this field. By adhering to these criteria, we aimed to capture the latest advancements and insights into genetic and epigenetic depression biomarkers among the broader array of depression biomarkers.

Throughout our methodology, meticulous attention was paid to ensuring the integrity and relevance of our findings. This involved not only the systematic search and selection of literature but also the critical evaluation and synthesis of data to provide a comprehensive overview. By rigorously following these methodological steps, including the application of the PICO framework, we aimed to contribute meaningfully to the understanding and advancement of knowledge regarding genetic and epigenetic depression biomarkers within the scientific community.

## 3. Results

The major step in our analytic approach involved article reviewing. Our results via article reviewing indicate firstly the diversity in the types of biomarkers (Table 1 [28]). In parallel to this, we discovered a growing interest in the biomarkers in psychiatry that represent information for the improvement of diagnosis, treatment by monitoring the volume of gray matter of the hippocampus [29], prefrontal cortex [30], basal ganglia through neuroimaging [31], presentation of HPA axis hyperactivity [32], thyroid gland dysfunction [33], monitoring of dopamine [34], noradrenaline [35], 5-HIAA [36], glutamate levels [37], monitoring of superoxide dismutase (SOD) activity [38] and lipid peroxidation [39], monitoring of adenosine levels of 3′,5′-monophosphate [40], activity of protein kinases [41], monitoring of pro-inflammatory cytokine levels [42], changes in tryptophan [43], kynurenine [44], insulin [45], and determination of gene polymorphisms [46]. Further, we found a cluster of studies that identified biomarkers that reflect the activity with potential to predict depression through the scope of inflammatory, neurotransmitter, neurotrophic, neuroendocrine, and metabolic systems origin (Table 2 [15]). They were identified to have the greatest potential in improving the diagnosis, treatment, and prevention of mental and physical health outcomes in individuals suffering from depression (Table 2 [15]).

**Figure 1:**
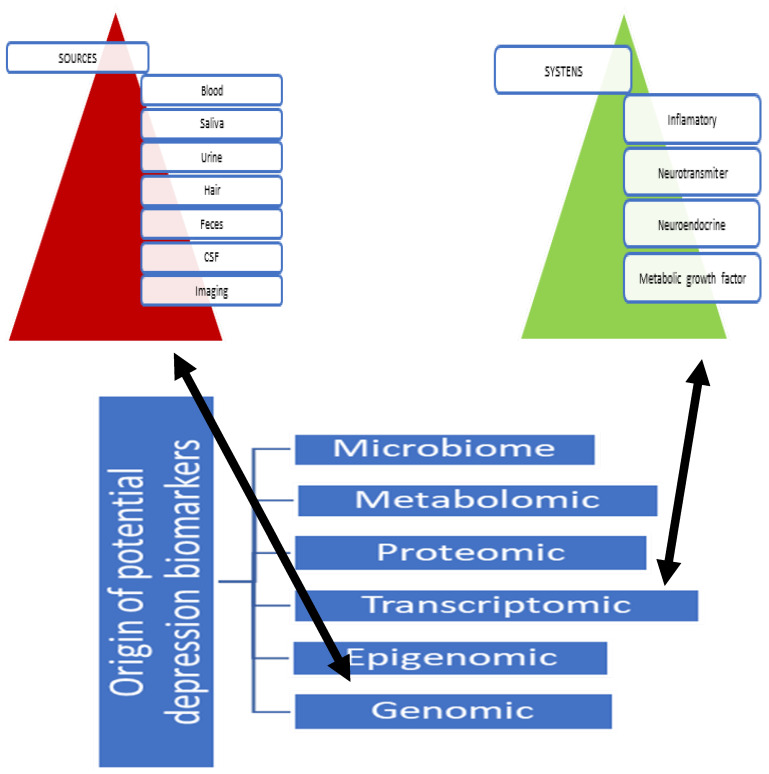
Potential biomarkers for depression: diverse biological levels including inflammatory markers, neurotransmitters, neuroendocrine factors, and growth factors, linked with various biological samples and imaging modalities across systems. Legend: genomic—refers to all genetic material in a human organism. Epigenomic or epigenome—refers to all changes to genetic material. Transcriptome—refers to all RNA transcripts from genetics. Proteoma—refers to all proteins expressed in a human organism. Metabolomic—refers to all small-molecule chemicals in a human organism. Microbiome—refers to all genes of microbes in an organism. Abrogation: CSF—cerebrospinal fluid. Source: Strawbridge, R., Young, A. H., & Cleare, A. J. (2017). Biomarkers for depression: recent insights, current challenges and future prospects. Neuropsychiatric disease and treatment, 13, 1245–1262. https://doi.org/10.2147/NDT.S114542 [15].

Evaluation of these systems through an omics approach revealed a wide range of biomarkers with translational potential. It also confirmed and reinforced the view that examining single, isolated factors is unlikely to provide data of clinical significance. A previously cited study proposed panels of biomarkers based on clinical and preclinical evidence. Based on the study’s proposed suggestions, we made Table 2 to illustrate these systems with input and overall dynamics, taking into account the source of biomarker potential sampling, the system of origin, and the level of human organism activity within which the biomarker functions. In other words, we illustrate the bidirectional causation between the functioning of diverse biological levels, including inflammatory markers, neurotransmitters, neuroendocrine factors, and growth factors, in relation to various modalities across systems in the human body detectable via presented biological samples and imaging (Figure 1 [15]).

After compiling and analyzing a diverse array of biomarkers—including diagnostic, therapeutic, prognostic, predictive, trait, and status markers—the results reveal compelling insights into their respective roles and implications. Each category offers unique perspectives on disease detection, treatment efficacy, patient prognosis, and more, shedding light on their potential applications in clinical practice. As we delve into these findings, the discussion will further explore their significance, limitations, and implications for future research and clinical applications.

## 4. Discussion

Molecular biomarkers hold significant promise for routine application in clinical psychiatry. Among psychiatric conditions, major depressive disorder (MDD) has garnered particular interest due to its rising prevalence and substantial impact on morbidity [47]. MDD is a biologically heterogeneous condition lacking reliable biomarkers to assess severity, subtypes, or treatment response. A growing body of evidence shows that alterations in peripheral growth factors, cytokines, endocrine, and metabolic markers not only reflect MDD pathophysiology but may also predict therapeutic outcomes—underscoring the need for a comprehensive biomarker panel to support diagnosis and personalized treatment approaches [48]. Depression is a multifactorial disorder arising from the interaction of genetic predisposition (nature) and environmental influences (nurture), both of which shape biomarker expression. Environmental factors such as stress, diet, trauma, and lifestyle can induce epigenetic changes—like DNA methylation, histone modification, and shifts in non-coding RNA expression—that affect gene activity without altering DNA sequences. Genetic factors, meanwhile, establish a baseline vulnerability, influencing brain function, neurotransmitter systems, and stress responses. Biomarkers often reflect the interplay between these genetic and epigenetic components: genetic variations predispose individuals to depression, while environmental factors modify gene expression and trigger disease onset. Understanding this interaction is key to advancing diagnostic and therapeutic strategies.

### 4.1. Inflammatory Biomarkers

In depression, inflammatory responses are consistently altered, even after adjusting for confounders such as BMI and age. Elevated inflammatory markers contribute to the onset and persistence of depressive episodes [49]. Stress, smoking, and obesity further elevate these markers, reinforcing their role as risk factors [50,51,52].

Most studies, including our initial findings (Table 1), confirm elevated CRP and IL-6 levels in depression. CRP levels correlate with treatment outcomes: patients with elevated CRP often do not respond to psychological therapies but respond better to pharmacological treatments like nortriptyline or fluoxetine, though not to escitalopram. Those with high CRP and treatment resistance show improved outcomes with infliximab (a TNFα antagonist) [53,54,55]. Elevated CRP is a reliable inflammatory marker linked to poor therapy response and increased risk of MDD onset and hospitalization [56]. IL-8 is also elevated in major depression [57], while IL-10 and interferon gamma levels vary between early responders and those with treatment resistance [57]. Remission is associated with reductions in IL-4 [58] and IL-2 [59]. Successful treatment generally lowers CRP, IL-6, IL-10, and IL-1β levels. TNFα decreases only in patients who respond well to therapy [59].

Chronic inflammation also promotes oxidative and nitrosative stress. This is marked by reduced levels of tryptophan, tyrosine, albumin, zinc, and HDL-cholesterol [60]. Oxidative stress biomarkers are detectable in blood, erythrocytes, mononuclear cells, urine, CSF, and brain tissue of depressed patients [61]. For example, uric acid levels drop in depression and increase with treatment, as do albumin, zinc, CoQ, and vitamin C [61].

### 4.2. Growth Factor–Neurogenic Biomarkers

Growth factors regulate neuronal development and survival. Though not inherently genetic or epigenetic, their expression is shaped by both, especially through environmental stressors. Therefore, changes in growth factor levels can signal underlying gene expression shifts relevant to depression.

BDNF (brain-derived neurotrophic factor) is the most studied neurogenic biomarker. Serum BDNF mirrors brain levels and is typically reduced in depressed patients, particularly in severe cases [62]. Treatment gradually increases BDNF, even in the absence of clinical improvement [63]. Conversely, pro-BDNF levels are elevated in depression but decline with treatment [63]. BDNF is also reduced in conditions like diabetes and smoking, both independent depression risk factors [63,64].

NGF (nerve growth factor) is consistently reduced in depression, correlating with symptom severity and remaining unchanged by treatment [65]. The same pattern applies to glial-derived neurotrophic factors. VEGF (vascular endothelial growth factor) is elevated in depressed patients, especially in treatment-resistant cases, likely due to inflammation and increased blood–brain barrier permeability [66]. IGF-1 and FGF-2 levels are also elevated and tend to decrease with treatment, though this contrasts with our findings [67,68].

### 4.3. Metabolic Biomarkers: Insights from AI

Metabolic biomarkers, such as glucose and lipid profiles, are influenced by epigenetic changes triggered by environmental factors, particularly through inflammatory and metabolic pathways. Metabolomic studies—now enhanced by AI—suggest that specific metabolite patterns in glucose-lipid signaling have strong predictive value for depression diagnosis [15].

In depressed individuals, metabolic alterations often occur regardless of comorbidities. Hyperglycemia, insulin resistance, and hypoalbuminemia are common, while leptin and ghrelin levels are typically low [69]. These levels generally normalize with treatment and remission.

### 4.4. Neurotransmitters as Biomarkers

Neurotransmitters, key regulators of mood and behavior, are influenced by both genetic and epigenetic factors. DNA methylation and histone modification can alter the expression of genes involved in neurotransmitter production, making these chemicals potential biomarkers for depression.

The monoamine theory, foundational to modern psychopharmacology, posits that depression stems from depleted serotonin, norepinephrine, and/or dopamine levels [70]. However, no neurotransmitter-based biomarker has proven clinically reliable for guiding treatment, largely because brain levels cannot be measured directly. Peripheral or urinary levels of metabolites are used as proxies, though they may not accurately reflect central concentrations. CSF provides more reliable measurements, and urine samples—especially when acidified—preserve molecule stability for liquid chromatography analysis [71].

Urinary noradrenaline (NA) correlates with depression and anxiety symptoms [72]. Low urinary 3-methoxy-4-hydroxyphenylglycol predicts positive response to NA-selective antidepressants like imipramine. As symptoms improve, levels of noradrenaline and dopamine metabolites (including 3-methoxy-4-hydroxyphenylglycol and homovanillic acid) typically rise. Interestingly, patients who respond well to SSRIs often exhibit lower levels of these metabolites [73].

Serotonin, often dubbed the “happiness hormone”, is assessed via 5-HIAA in CSF and urine. Decreased levels are more closely linked to impulsivity, aggression, and suicidality than to depression itself [74]. Serotonin’s effects are mediated primarily through 5-HT1A receptors, which regulate neuronal activity both pre- and post-synaptically [75]. An exaggerated autoimmune response to serotonin is linked to recurrent depressive episodes, while increased density of 5-HT2A receptors on platelets has been associated with suicidality and may serve as a risk marker [76,77].

Dopamine activity is also diminished in untreated or early-stage depression [34]. Glutamatergic dysfunction in depression is marked by elevated glutamate and GABA levels, often in conjunction with reduced 5-HT and NA levels [78,79].

### 4.5. Neuroendocrine Biomarkers of the HPA Axis

The neuroendocrine system, particularly the hypothalamic–pituitary–adrenal (HPA) axis, plays a central role in stress response. While primarily regulated genetically, its sensitivity and reactivity can be shaped by epigenetic modifications, especially under chronic stress. As such, HPA axis biomarkers reflect both genetic vulnerability and environmental influence.

Depression is commonly linked to HPA axis dysregulation, typically presenting with elevated basal cortisol, impaired suppression in the dexamethasone suppression test, and sustained elevation of corticotropin-releasing hormone. Glucocorticoids, the primary stress hormones, impact hippocampal neurogenesis. Thyroid and sex hormones (e.g., estrogen, testosterone) have also been implicated [80].

Cortisol is the most studied HPA axis biomarker, measurable in blood, urine, saliva, and hair. Hypercortisolemia is consistently observed in depression and aligns with findings from the first stage of our research. Hair cortisol reflects chronic stress exposure and helps differentiate depression from other psychiatric disorders [81]. Salivary cortisol levels, particularly after waking, have shown diagnostic value in adolescents [82].

Elevated cortisol predicts poor response to both psychological and pharmacological therapies [83]. In contrast, patients with a history of childhood trauma may exhibit blunted cortisol reactivity. Atypical depression is associated with reduced cortisol levels, which may aid in distinguishing it from melancholic depression and explain heightened sensitivity to reward and rejection.

### 4.6. Biomarkers in Predicting Treatment Response

The dexamethasone suppression test has historically been the most investigated tool for predicting treatment outcomes, especially in melancholic depression [84]. Lack of cortisol suppression indicates reduced likelihood of remission, though its clinical utility remains limited due to inadequate sensitivity and specificity. A shift from non-suppression to suppression during treatment suggests therapeutic effectiveness, while an increased cortisol response post-treatment may predict relapse risk [84].

Other hormones, including corticotropin-releasing hormone, adrenocorticotropin, and vasopressin, are elevated in depression, though findings are inconsistent. Notably, corticotropin-releasing hormone and its mRNA are elevated in the brains of suicide victims with depression [85]. Conversely, dehydroepiandrosterone (DHEA) levels are typically reduced [86], and the cortisol-to-DHEA ratio is elevated in treatment-resistant depression, even during remission [87].

### 4.7. Trait Biomarkers

Trait biomarkers identify individuals predisposed to depression [28] (Table 2). For example, abnormal TSH levels, seen in trending or established hypothyroidism, may contribute to depressive disorders, as supported by our initial research findings (Table 1) [88]. Treating depression may concurrently improve thyroid function.

Depressed individuals often have reduced nocturnal melatonin and a delayed daytime peak. Evidence suggests hormone replacement therapy enhances antidepressant efficacy, as thyroid hormones appear to support recovery during treatment [89].

### 4.8. Signaling Pathways Modified by Antidepressants

Signaling pathways such as glycogen synthase kinase-3 (GSK-3), mitogen-activated protein kinase (MAPK), and phosphoinositide 3-kinase (PI3K) are believed to play a key role in treatment mechanisms. These pathways are potential biomarkers for therapeutic response [90], though further research is needed to validate their clinical relevance.

### 4.9. Genetic and Epigenetic Factors in Depression

Both genetic and epigenetic factors contribute significantly to depression. Genetic predispositions involve inherited DNA variations that affect neurotransmission, inflammation, and stress regulation. Epigenetic modifications—such as DNA methylation and histone alteration—are shaped by environmental exposures and life experiences, influencing gene expression without altering the DNA sequence.

Key gene–environment interactions, including those involving serotonin transporter (SLC6A4) and inflammatory genes (e.g., TNF-α, IL-6), have been implicated in depression. Epigenetic modifications of these genes can influence their activity and increase susceptibility [91]. Early life stress can cause enduring epigenetic changes, increasing the long-term risk for depression and illustrating how the environment modulates genetic risk.

### 4.10. Biomarkers Linking Genetics, Epigenetics, and Treatment Response

Currently, major depressive disorder (MDD) is diagnosed through clinical evaluation and self-reported symptoms, with no objective or non-invasive tests available. The identification of biomarkers—such as inflammatory markers, neurotrophic factors, hormones, genetic and epigenetic profiles, and neuroimaging findings—holds promise for improving diagnosis, guiding treatment selection, and predicting therapeutic response. While research is still evolving, biomarkers like BDNF, HPA axis activity, cytokines, and emerging epigenetic insights show particular potential in advancing personalized care for MDD [92]. Biomarkers that integrate genetic and epigenetic factors hold promise for predicting treatment outcomes. Genetic polymorphisms in drug-metabolizing enzymes, such as cytochrome P450 variants, help forecast antidepressant efficacy and side effects. Similarly, epigenetic markers—like methylation patterns in stress-related genes (e.g., FKBP5)—may signal treatment resistance or susceptibility [93].

These tools support personalized medicine by allowing treatment plans tailored to an individual’s genetic and epigenetic profile. For example, patients with certain 5-HTT gene variants may respond better to SSRIs, while others might benefit more from psychotherapy or neuromodulation techniques like deep brain stimulation [94].

Moreover, therapies targeting epigenetic reprogramming are gaining attention. Agents like ketamine can rapidly induce epigenetic changes in brain regions responsible for mood regulation, enhancing synaptic plasticity and stress resilience. Understanding the dynamic between genetic predisposition and epigenetic modulation is key to developing more effective, individualized treatments.

### 4.11. Summary of Depression Biomarkers—Limitations of Current Research

Numerous biomarkers are associated with depression, though their roles vary—some predict treatment response, others guide therapy selection, and many change during treatment independent of outcome. However, current evidence is insufficient to form a cohesive, clinically applicable model.

Some biomarkers are epiphenomenal or relevant only in specific subgroups. Genetic and epigenetic markers are gaining prominence, with genetic variants like 5-HTTLPR reflecting inherited vulnerability, and epigenetic changes (e.g., DNA methylation, histone modifications) showing how environmental factors modulate gene expression.

Research challenges include inter-individual variability and lack of standardization. While proteomic biomarkers show potential, genomic and epigenetic factors are more difficult to study due to environmental influences. For example, early life stress can epigenetically alter FKBP5 expression, potentially affecting subsequent generations despite unchanged DNA sequences.

### 4.12. Clinical Implications—Strengths of Current Research

Genetic and epigenetic biomarkers could transform depression treatment through personalized medicine. Identifying specific genetic variants may enable clinicians to match patients with the most effective antidepressants, reducing the need for trial-and-error prescribing.

Epigenetic markers can also reveal how life experiences affect gene expression, guiding early intervention strategies. Epigenetic therapies may help reverse or mitigate stress-related biological changes, offering novel treatment approaches beyond symptom relief.

Because epigenetic changes are modifiable, they are especially valuable for tailoring treatment. If a patient’s epigenetic profile predicts poor response to a medication, alternative therapies can be pursued earlier, improving recovery prospects.

### 4.13. Future Research Directions

As research advances, genetic and epigenetic markers will play a growing role in understanding and treating depression. Future studies should focus on how gene–environment interactions shape epigenetic profiles and contribute to depression risk. Although some genetic loci related to serotonin and dopamine have been identified, their individual effects are small and likely require complementary epigenetic changes for expression. Epigenetic mechanisms—such as DNA methylation, histone modification, and non-coding RNA activity—are essential in mediating gene–environment interactions. Stress, especially during critical developmental periods, can cause long-lasting epigenetic modifications that alter HPA axis functioning and mood regulation, increasing depression risk. Integrating genomic and epigenomic analyses will be vital in identifying novel biomarkers. Longitudinal studies tracking these changes over time are necessary for translating findings into clinical practice. Additionally, epigenetic therapies, including drugs targeting methylation or non-coding RNAs, could provide innovative treatment options for depression. Got it! Here is a refined version that clearly states that while the area has been well-studied, the evidence so far has not been robust or conclusive, and highlights the direction future research should take [95]. Previous studies identified that abnormalities in brain-derived neurotrophic factor (BDNF) levels, elevated endocrine markers such as cortisol, thyroid-stimulating hormone (TSH), and prolactin, as well as structural brain changes observed through neuroimaging—including reduced hippocampal volume and cortical thinning—are consistently associated with major depressive disorder (MDD). These biomarkers reflect underlying neurobiological alterations linked to the disorder, offering valuable insight into its pathophysiology. Their clinical relevance lies in the potential to improve diagnostic precision and support the development of more individualized and effective treatment strategies for those affected by MDD [96].

Although major depressive disorder (MDD) has been extensively studied through the lens of various biological theories—including neuroimaging, immune function, hormone dysregulation, neurotransmission, and oxidative stress—the existing body of prospective research has not yielded consistently robust or conclusive biomarkers. Despite considerable investigation, most findings across these domains have been either statistically insignificant or too heterogeneous to synthesize meaningfully. Cortisol has shown some potential as a predictive marker, but its reliability is compromised by confounding factors such as pre-existing clinical conditions. These outcomes highlight a clear gap between theoretical models and empirical validation, underscoring the need for future research to prioritize standardized methodologies, larger sample sizes, and more rigorous longitudinal designs in order to establish reliable biomarkers for the onset, recurrence, and treatment response of MDD.

### 4.14. Challenges in Translating Biomarkers into Clinical Practice

This section elaborates on the limitations and hurdles that currently hinder the routine clinical implementation of biomarkers, despite the growing body of promising evidence. Specifically, we explore the variability in laboratory methods, the lack of standardized protocols across studies, and the absence of consensus on clinically relevant thresholds for many biomarkers. We also discuss the financial and infrastructural barriers faced by many healthcare systems, particularly in resource-limited settings, which make widespread biomarker testing impractical at this stage.

Additionally, we address the issue of biomarker accessibility and equity. Many of the advanced tests, especially those involving genomic or epigenomic profiling, remain largely restricted to specialized research centers and are not yet integrated into primary care or routine psychiatric assessment. This creates a gap between research advancements and everyday clinical practice.

By incorporating this critical discussion, we aim to present a more balanced and realistic view of the current state of biomarker research in depression. We thank the reviewer for helping to highlight this essential dimension, and we believe that the revised manuscript now offers a more complete and practically relevant analysis of this evolving field.

We are grateful to the reviewer for highlighting the need for a more critical and analytical evaluation of the current biomarker literature. This is an especially important observation, as much of the existing research—while rich in promise—is also marked by methodological inconsistencies, heterogeneous findings, and limited translational applicability.

### 4.15. Critical Appraisal of Biomarker Literature in the Current Review: Contradictions, Limitations, and Interpretative Challenges

While biomarker research in depression holds considerable potential, the literature is marked by inconsistencies, contradictions, and methodological limitations that complicate translation into clinical practice. A nuanced appraisal of these challenges is essential for a realistic understanding of the field’s current state.

In the domain of inflammatory biomarkers, markers such as CRP and IL-6 have shown consistent elevation in depressed patients [49,50,51,52,53,54,55]. However, cytokines like IL-10 and interferon gamma yield inconsistent patterns across studies [57], suggesting that inflammation in depression may vary significantly between subtypes or individuals. This variability challenges the idea of a universal inflammatory signature in depression.

Similarly, while BDNF is widely reported to be reduced in depression and increased with treatment [62], recent findings indicate BDNF may rise independently of clinical response [63], questioning its utility as a dynamic marker of therapeutic progress. Further complexity arises with neurotrophins like NGF and glial-derived factors, which appear unaffected by treatment [65], underlining the need for subtype-specific biomarker models.

The monoamine hypothesis—central to early antidepressant development—remains controversial. Peripheral measurements of neurotransmitter metabolites (e.g., in urine or CSF) do correlate with symptom changes and treatment response [72,73], yet their indirect nature limits the ability to draw firm conclusions about central nervous system activity [71]. For example, while serotonin receptor alterations are linked to suicidality [75,76,77], their specificity for depression diagnosis remains unclear.

Metabolic and HPA axis biomarkers, including cortisol, show robust associations with depression [80,81,82,83], but are influenced by numerous external factors such as obesity, circadian rhythm, and early life stress, leading to conflicting findings—especially in atypical depression, where cortisol levels may be paradoxically low.

Methodologically, many studies suffer from small sample sizes, inadequate controls, and non-standardized protocols. Cross-sectional designs predominate, limiting the ability to infer causality or assess longitudinal changes. The use of peripheral biomarkers to infer central changes is another major limitation. Genetic and epigenetic research, while advancing, is still hampered by replication challenges and population-specific effects. For instance, findings related to the FKBP5 gene and 5-HTTLPR polymorphism [91,93,94] are often dependent on specific environmental interactions, such as early life trauma, and may not generalize broadly.

Altogether, the current body of literature reflects a field in transition—scientifically rich, yet methodologically fragmented. For biomarkers to achieve clinical relevance, future studies must adopt larger, longitudinal, and multi-site designs, incorporate standardized assays, and stratify subjects by depression subtype and comorbidities. Only through such rigorous frameworks can the field move beyond correlation and toward actionable, individualized psychiatry.

## 5. Conclusions

Identifying biomarkers associated with depressive disorders, particularly those rooted in genetic and epigenetic factors, represents a critical step toward improving both diagnostic precision and treatment efficacy. As depression manifests differently across individuals, the ability to pinpoint specific genetic variations and epigenetic modifications that underlie these diverse presentations is essential for developing personalized therapeutic approaches. By understanding the complex biological underpinnings of depression, this study contributes to the ongoing efforts to create targeted treatments that address the unique needs of individuals affected by this debilitating group of disorders.

Current research has revealed that genetic predispositions—such as mutations or polymorphisms in genes related to neurotransmitter systems—are key contributors to an individual’s risk of developing depression. However, epigenetic modifications, which can alter gene expression in response to environmental factors like stress, trauma, or lifestyle, add an additional layer of complexity. These epigenetic markers may explain why depression can affect individuals differently, even those with similar genetic backgrounds. Furthermore, understanding how epigenetic changes can be modulated by therapeutic interventions offers exciting possibilities for new treatment modalities that go beyond traditional antidepressants.

To fully realize the potential of genetic and epigenetic biomarkers in clinical settings, future research must prioritize validating these markers across larger, more diverse cohorts. Longitudinal studies are crucial for understanding how these biomarkers evolve over time and in response to treatments. Large-scale clinical trials will be essential for establishing the clinical relevance of genetic and epigenetic biomarkers and for determining their predictive value in terms of treatment response, relapse, and long-term recovery.

The promise of genetic and epigenetic biomarkers extends beyond diagnostics and treatment. These biomarkers may also contribute to the earlier detection of depression, enabling clinicians to intervene before the condition progresses to more severe stages. Moreover, they can help predict individual responses to various therapies, guiding clinicians in selecting the most effective treatment strategies tailored to each patient’s unique biological profile. Ultimately, the integration of genetic and epigenetic biomarkers into clinical practice will lead to improved patient outcomes, providing a more personalized and effective approach to managing depression.

In conclusion, while much work remains to be done, the future of depression treatment lies in a deeper understanding of the genetic and epigenetic factors that contribute to the disorder. By validating and integrating these biomarkers into clinical practice, researchers and clinicians can begin to address the complex biological landscape of depression in a way that allows for more accurate diagnoses and more effective, personalized treatment strategies.

## Figures and Tables

**Table 1:** Comprehensive overview of diverse biomarker types: type of biomarker, its purpose, examples, and how it is applied in clinical practice.

Type of Biomarkers	Purpose/Use	Examples	Clinical Application
**Diagnostic Biomarkers**	Confirm the presence or absence of disease	Genetic mutations, specific proteins	Early detection of diseases like cancer, infectious diseases
**Markers of Therapy**	Help choose the optimal therapy for the patient	Tumor markers, genetic profiling	Personalizing treatment in oncology, choosing targeted therapies
**Therapy Mediator**	Monitor the response to therapy	Blood levels of drugs, specific biomarkers	Monitoring therapy effectiveness, adjusting dosages in chronic diseases
**Prognostic Markers**	Predict the course or progression of the disease	Gene expression profiles, protein levels	Prognosis of cancer, cardiovascular diseases, or chronic illnesses
**Predictive Markers**	Predict the likelihood of disease development	Genetic risk factors, biomarkers of predisposition	Predicting risk of developing diseases like diabetes, Alzheimer’s
**Trait Markers**	Identify individuals with a higher risk due to genetic factors	SNPs, familial risk factors	Identifying genetic predispositions to hereditary conditions
**Status Markers**	Reflect the current clinical status of the patient	Inflammatory markers, viral load	Assessing disease activity, infection status, or inflammation levels

Source: Lopresti, A. L., Maker, G. L., Hood, S. D., & Drummond, P. D. (2014). A review of peripheral biomarkers in major depression: the potential of inflammatory and oxidative stress biomarkers. Progress in neuro-psychopharmacology & biological psychiatry, 48, 102–111. https://doi.org/10.1016/j.pnpbp.2013.09.017 [28].

**Table 2:** Comprehensive insights into diverse origins of depression biomarkers: classification across systems of inflammation, neuroendocrine, growth factors, and neurotransmitters.

Biomarker System	Key Findings	Effect/Impact	Therapeutic Relevance
**Inflammation Biomarkers**	Higher levels of pro-inflammatory markers in patients with depression compared to controls. Antidepressants reduce inflammation levels.	Inflammation is higher in depression, indicating an immune system imbalance.	Anti-inflammatory therapy leads to improvements in depressive symptoms.
**Neuroendocrine Biomarkers**	HPA axis hyperactivity in depressed patients, resulting in hypercortisolism. High cortisol levels correlate with poor response to therapy.	Elevated cortisol indicates stress and poor treatment response.	Targeting HPA axis dysregulation may improve therapeutic outcomes.
**Growth Factor Biomarkers**	Lower neurotrophic factor levels (e.g., BDNF, NGF) in depressed patients compared to controls. These factors increase with therapy, regardless of symptom improvement.	Decreased neurotrophic factors may contribute to depression.	Monitoring neurotrophic factor levels may provide insights into therapeutic progress.
**Neurotransmitter Biomarkers**	Increased binding to 5-HT1A receptors in depressed individuals. Monoamine interaction affects cognitive function and stress response.	Serotonin receptor activity influences mood and cognitive resources.	Targeting serotonin receptors can optimize treatment response and manage therapeutic resistance.
**Metabolic Biomarkers**	Depression linked with altered metabolic profiles. BMI and disease severity influence these factors. Atypical depression forms often show metabolic disorders.	Metabolic imbalances complicate depression and therapy.	Metabolic profile assessment can guide treatment, particularly for atypical depression forms.

Source: Strawbridge, R., Young, A. H., & Cleare, A. J. (2017). Biomarkers for depression: recent insights, current challenges and future prospects. Neuropsychiatric disease and treatment, 13, 1245–1262. https://doi.org/10.2147/NDT.S114542 [15].

## Data Availability

The data used in this review can be obtained upon request corresponding author.

## Abbreviations

CRP	C-reactive protein
TNFα	Tumor Necrosis Factor α
IL-l b	Interleukin 1 β
IL-2	Interleukin 2
IL-4	Interleukin 4
IL-10	Interleukin 10
IFNγ	Interferon γ or type II interferon
IL-8	Interleukin 8
MCP4	Human monocyte chemoattractant protein
IL-1a	Interleukin-1 α
IFNα	Interferon α
IL-5	Interleukin 8
IL-7	Interleukin 7
IL-12	Interleukin 12
IL-12p70	Interleukin-12p70
IL-13	Interleukin 13
IL-15	Interleukin 15
IL-16	Interleukin 16
IL-17	Interleukin 17
TNFβ	Tumor necrosis factor β
Mipla	Methylisopropyllysergamide a
Miplb	Methylisopropyllysergamide b
SAA	*Serum Amyloid A*
sICAMI	Soluble intercellular adhesion molecule-1
sVCAMI	Soluble vascular cell adhesion molecule-1
TARC	Thymus and activation-regulated chemokine
IP-10	Interferon γ-induced protein 10
GM-CSF	Granulocyte Macrophage Colony-Stimulating Factor
BDNF	Brain-Derived Neurotrophic Factor
VEGF	Vascular endothelial growth factor
NGF	Nerve growth factor
GDNF	Glial cell line-derived neurotrophic factor
IGF-l	Expression of Insulin-Like Growth Factor-I
bFGF	Basic Fibroblast Growth Factor
Tie2	Tyrosine kinases receptor 2
PIGF	Phosphatidylinositol Glycan Anchor Biosynthesis Class F
VEGFC	*Vascular endothelial growth factor C*
VEGFD	*Vascular endothelial growth factor D*
proBDNF	Precursor of Brain-Derived Neurotrophic Factor
5-HT	*5-hydroxytryptamine* receptors, or serotonin receptors
NA	*Noradrenalin*
DA	Dopamine
GABA	γ-Aminobutyric Acid
MHPG	3-Methoxy-4-hydroxyphenylglycol
HVA	Homovanillic acid
ACTH	Adrenokortikotropni hormone
CRH	Corticotropin-releasing hormone
DHEA	Dehydroepiandrosterone
TSH	Thyroid stimulating hormone

## References

[1] Milic J., Stankic D., Stefanovic D., Patel V.B., Preedy V.R. (2023). Eating Disorder and Quality of Life. Eating Disorders.

[2] World Health Organization (2002). The World Health Report 2002-Reducing Risks, Promoting Healthy Life.

[3] Baig-Ward K.M., Jha M.K., Trivedi M.H. (2023). The Individual and Societal Burden of Treatment-Resistant Depression: An Overview. Psychiatr. Clin. N. Am..

[4] Gonda X., Fountoulakis K.N., Kaprinis G., Rihmer Z. (2007). Prediction and prevention of suicide in patients with unipolar depression and anxiety. Ann. Gen. Psychiatry.

[5] Greenberg P.E., Fournier A.A., Sisitsky T., Pike C.T., Kessler R.C. (2015). The economic burden of adults with major depressive disorder in the United States (2005 and 2010). J. Clin. Psychiatry.

[6] Greenberg P., Fournier A.-A., Sisitsky T., Simes M., Berman R., Koenigsberg S.H., Kessler R.C. (2021). The economic burden of adults with major depressive disorder in the United States (2010 and 2018). PharmacoEconomics.

[7] (2021). Anxiety and Depression. https://www.cdc.gov/nchs/covid19/pulse/mental-health.htm.

[8] Centers for Disease Control and Prevention (2020). Early Release of Selected Mental Health Estimates Based on Data from the January–June 2019 National Health Interview Survey.

[9] Zhang X., Zhang Z., Diao W., Zhou C., Song Y., Wang R., Luo X., Liu G. (2023). Early diagnosis of major depressive disorder: From biomarkers to point-of-care testing. TrAC Trends Anal. Chem..

[10] Chan V.K.Y., Leung M.Y.M., Chan S.S.M., Yang D., Knapp M., Luo H., Craig D., Chen Y., Bishai D.M., Wong G.H.Y. (2024). Projecting the 10-year costs of care and mortality burden of depression until 2032: A Markov modelling study developed from real-world data. The Lancet regional health. West. Pac..

[11] Wamala S.P., Lynch J., Horsten M., Mittleman M.A., Schenck-Gustafsson K., Orth-Gomer K. (1999). Education and the metabolic syndrome in women. Diabetes Care.

[12] Mojtabai R., Amin-Esmaeili M., Spivak S., Olfson M. (2021). Remission and Treatment Augmentation of Depression in the United States. J. Clin. Psychiatry.

[13] Ratnayake S. Why Has the Misleading “Chemical Imbalance” Theory of Mental Illness Persisted for So Long? State of Mind. Slate. 4 August 2022. https://slate.com/technology/2022/08/ssris-chemical-imbalance-depression.html.

[14] Singhal N. Stigma, Prejudice and Discrimination Against People with Mental Illness: A Physician Review; American Psychiatric Association Portal of Patients and Families. March 2024. https://www.psychiatry.org/patients-families/stigma-and-discrimination.

[15] Strawbridge R., Young A.H., Cleare A.J. (2017). Biomarkers for depression: Recent insights, current challenges and future prospects. Neuropsychiatr. Dis. Treat..

[16] Paganin W., Signorini S. (2024). Inflammatory biomarkers in depression: Scoping review. BJPsych Open.

[17] Dunlop B.W., Mayberg H.S. (2017). Neuroimaging Advances for Depression. Cerebrum Dana Forum Brain Sci..

[18] Licinio J., Wong M.L. (2020). Advances in depression research: Second special issue, 2020, with highlights on biological mechanisms, clinical features, co-morbidity, genetics, imaging, and treatment. Mol. Psychiatry.

[19] Coleman J.R.I., Peyrot W.J., Purves K.L., Davis K.A.S., Rayner C., Choi S.W., Hübel C., Gaspar H.A., Kan C., Van der Auwera S. (2020). Genome-wide gene-environment analyses of major depressive disorder and reported lifetime traumatic experiences in UK Biobank. Mol. Psychiatry.

[20] Amare A.T., Vaez A., Hsu Y.-H., Direk N., Kamali Z., Howard D.M., McIntosh A.M., Tiemeier H., Bültmann U., Snieder H. (2019). Bivariate genome-wide association analyses of the broad depression phenotype combined with major depressive disorder, bipolar disorder or schizophrenia reveal eight novel genetic loci for depression. Mol. Psychiatry.

[21] Li J.M., Jiang C.L. (2020). Biological Diagnosis of Depression: A Biomarker Panel from Several Nonspecial Indicators Instead of the Specific Biomarker(s). Neuropsychiatr. Dis. Treat..

[22] Taub D.D. (2008). Neuroendocrine interactions in the immune system. Cell. Immunol..

[23] Guo X.J., Wu P., Jia X., Dong Y.M., Zhao C.M., Chen N.N., Zhang Z.Y., Miao Y.T., Yun K.M., Gao C.R. (2022). Mapping the structure of depression biomarker research: A bibliometric analysis. Front. Psychiatry.

[24] Lima Giacobbo B., Doorduin J., Klein H.C., Dierckx R.A.J.O., Bromberg E., de Vries E.F.J. (2019). Brain-Derived Neurotrophic Factor in Brain Disorders: Focus on Neuroinflammation. Mol. Neurobiol..

[25] Felger J.C., Lotrich F.E. (2013). Inflammatory cytokines in depression: Neurobiological mechanisms and therapeutic implications. Neuroscience.

[26] Beer C., Rae F., Semmler A., Voisey J. (2024). Biomarkers in the Diagnosis and Prediction of Medication Response in Depression and the Role of Nutraceuticals. Int. J. Mol. Sci..

[27] Malik S., Singh R., Arora G., Dangol A., Goyal S. (2021). Biomarkers of Major Depressive Disorder: Knowing is Half the Battle. Clin. Psychopharmacol. Neurosci..

[28] Lopresti A.L., Maker G.L., Hood S.D., Drummond P.D. (2014). A review of peripheral biomarkers in major depression: The potential of inflammatory and oxidative stress biomarkers. Prog. Neuropsychopharmacol. Biol. Psychiatry.

[29] Peterson B.S., Kaur T., Baez M.A., Whiteman R.C., Sawardekar S., Sanchez-Peña J., Hao X., Klahr K.W., Talati A., Wickramaratne P. (2022). Morphological Biomarkers in the Amygdala and Hippocampus of Children and Adults at High Familial Risk for Depression. Diagnostics.

[30] Pizzagalli D.A., Roberts A.C. (2022). Prefrontal cortex and depression. Neuropsychopharmacology.

[31] Wang K., Hu Y., Yan C., Li M., Wu Y., Qiu J., Zhu X., the REST-meta-MDD Consortium (2023). Brain structural abnormalities in adult major depressive disorder revealed by voxel- and source-based morphometry: Evidence from the REST-meta-MDD Consortium. Psychol. Med..

[32] Iob E., Kirschbaum C., Steptoe A. (2020). Persistent depressive symptoms, HPA-axis hyperactivity, and inflammation: The role of cognitive-affective and somatic symptoms. Mol. Psychiatry.

[33] Hage M.P., Azar S.T. (2012). The Link between Thyroid Function and Depression. J. Thyroid Res..

[34] Cui L., Li S., Wang S., Wu X., Liu Y., Yu W., Wang Y., Tang Y., Xia M., Li B. (2024). Major depressive disorder: Hypothesis, mechanism, prevention and treatment. Signal Transduct. Target. Ther..

[35] Kurita M., Nishino S., Numata Y., Okubo Y., Sato T. (2015). The noradrenaline metabolite MHPG is a candidate biomarker between the depressive, remission, and manic states in bipolar disorder I: Two long-term naturalistic case reports. Neuropsychiatr. Dis. Treat..

[36] Jayamohananan H., Manoj Kumar M.K., P A.T. (2019). 5-HIAA as a Potential Biological Marker for Neurological and Psychiatric Disorders. Adv. Pharm. Bull..

[37] Khoodoruth M.A.S., Estudillo-Guerra M.A., Pacheco-Barrios K., Nyundo A., Chapa-Koloffon G., Ouanes S. (2022). Glutamatergic System in Depression and Its Role in Neuromodulatory Techniques Optimization. Front. Psychiatry.

[38] Stefanescu C., Ciobica A. (2012). The relevance of oxidative stress status in first episode and recurrent depression. J. Affect. Disord..

[39] Ait Tayeb A.E.K., Poinsignon V., Chappell K., Bouligand J., Becquemont L., Verstuyft C. (2023). Major Depressive Disorder and Oxidative Stress: A Review of Peripheral and Genetic Biomarkers According to Clinical Characteristics and Disease Stages. Antioxidants.

[40] Bartoli F., Burnstock G., Crocamo C., Carrà G. (2020). Purinergic Signaling and Related Biomarkers in Depression. Brain Sci..

[41] Pandey G.N., Sharma A., Rizavi H.S., Ren X. (2021). Dysregulation of Protein Kinase C in Adult Depression and Suicide: Evidence from Postmortem Brain Studies. Int. J. Neuropsychopharmacol..

[42] Harsanyi S., Kupcova I., Danisovic L., Klein M. (2022). Selected Biomarkers of Depression: What Are the Effects of Cytokines and Inflammation?. Int. J. Mol. Sci..

[43] Correia A.S., Vale N. (2022). Tryptophan Metabolism in Depression: A Narrative Review with a Focus on Serotonin and Kynurenine Pathways. Int. J. Mol. Sci..

[44] Zong L., Ge M., Wang J., Kuang D., Wei H., Wang Z., Hu Z., Zhao C., Jin Q., Chen M. (2024). Causal association between kynurenine and depression investigated using two-sample mendelian randomization. Sci. Rep..

[45] Fernandes B.S., Salagre E., Enduru N., Grande I., Vieta E., Zhao Z. (2022). Insulin resistance in depression: A large meta-analysis of metabolic parameters and variation. Neurosci. Biobehav. Rev..

[46] Nunes F.D.D., Ferezin L.P., Pereira S.C., Figaro-Drumond F.V., Pinheiro L.C., Menezes I.C., Baes C.V.W., Coeli-Lacchini F.B., Tanus-Santos J.E., Juruena M.F. (2022). The Association of Biochemical and Genetic Biomarkers in VEGF Pathway with Depression. Pharmaceutics.

[47] Galvão A.C.d.M., Almeida R.N., Júnior G.M.d.S., Leocadio-Miguel M.A., Palhano-Fontes F., de Araujo D.B., Lobão-Soares B., Maia-De-Oliveira J.P., Nunes E.A., Hallak J.E.C. (2021). Potential biomarkers of major depression diagnosis and chronicity. PLoS ONE.

[48] Schmidt H.D., Shelton R.C., Duman R.S. (2011). Functional biomarkers of depression: Diagnosis, treatment, and pathophysiology. Neuropsychopharmacology.

[49] Shi R., Gwee X., Chua D.Q., Tan C.T., Yap K.B., Larbi A., Lu Y., Ng T.P. (2022). Inflammatory markers and incident depression: Evidence in a population-based prospective study. Psychoneuroendocrinology.

[50] Asthana A., Johnson H.M., Piper M.E., Fiore M.C., Baker T.B., Stein J.H. (2010). Effects of smoking intensity and cessation on inflammatory markers in a large cohort of active smokers. Am. Heart J..

[51] Ellulu M.S., Patimah I., Khazáai H., Rahmat A., Abed Y. (2017). Obesity and inflammation: The linking mechanism and the complications. Arch. Med. Sci..

[52] Kühnel A., Hagenberg J., Knauer-Arloth J., Ködel M., Czisch M., Sämann P.G., Brückl T., Spoormaker V.I., Erhardt A., BeCOME Working Group (2023). Stress-induced brain responses are associated with BMI in women. Commun. Biol..

[53] Jha M.K., Minhajuddin A., Gadad B.S., Greer T., Grannemann B., Soyombo A., Mayes T.L., Rush A.J., Trivedi M.H. (2017). Can C-reactive protein inform antidepressant medication selection in depressed outpatients? Findings from the CO-MED trial. Psychoneuroendocrinology.

[54] Orsolini L., Pompili S., Tempia Valenta S., Salvi V., Volpe U. (2022). C-Reactive Protein as a Biomarker for Major Depressive Disorder?. Int. J. Mol. Sci..

[55] Pan Y., Luo R., Zhang S., Liu Y., Wang Y., Feng S., Li H. (2022). C-reactive protein could predict the efficacy of SSRIs in clinical practice: A cohort study of large samples in the real world. J. Affect. Disord..

[56] Wium-Andersen M.K., Ørsted D.D., Nielsen S.F., Nordestgaard B.G. (2013). Elevated C-Reactive Protein Levels, Psychological Distress, and Depression in 73 131 Individuals. JAMA Psychiatry.

[57] Zincir S., Öztürk P., Bilgen A.E., İzci F., Yükselir C. (2016). Levels of serum immunomodulators and alterations with electroconvulsive therapy in treatment-resistant major depression. Neuropsychiatr. Dis. Treat..

[58] Lu L., Hu X., Jin X. (2023). IL-4 as a potential biomarker for differentiating major depressive disorder from bipolar depression. Medicine.

[59] Poletti S., Zanardi R., Mandelli A., Aggio V., Finardi A., Lorenzi C., Borsellino G., Carminati M., Manfredi E., Tomasi E. (2024). Low-dose interleukin 2 antidepressant potentiation in unipolar and bipolar depression: Safety, efficacy, and immunological biomarkers. Brain Behav. Immun..

[60] Yao L., Pan L., Qian M., Sun W., Gu C., Chen L., Tang X., Hu Y., Xu L., Wei Y. (2020). Tumor Necrosis Factor-α Variations in Patients with Major Depressive Disorder Before and After Antidepressant Treatment. Front. Psychiatry.

[61] Vaváková M., Ďuračková Z., Trebatická J. (2015). Markers of Oxidative Stress and Neuroprogression in Depression Disorder. Oxidative Med. Cell. Longev..

[62] Autry A.E., Monteggia L.M. (2012). Brain-derived neurotrophic factor and neuropsychiatric disorders. Pharmacol. Rev..

[63] Monteiro B.C., Monteiro S., Candida M., Adler N., Paes F., Rocha N., Nardi A.E., Murillo-Rodriguez E., Machado S. (2017). Relationship Between Brain-Derived Neurotrofic Factor (Bdnf) and Sleep on Depression: A Critical Review. Clin. Pract. Epidemiol. Ment. Health CP EMH.

[64] Xia H., Du X., Yin G., Zhang Y., Li X., Cai J., Huang X., Ning Y., Soares J.C., Wu F. (2019). Effects of smoking on cognition and BDNF levels in a male Chinese population: Relationship with BDNF Val66Met polymorphism. Sci. Rep..

[65] Maes M., Rachayon M., Jirakran K., Sodsai P., Sughondhabirom A. (2023). Lower Nerve Growth Factor Levels in Major Depression and Suicidal Behaviors: Effects of Adverse Childhood Experiences and Recurrence of Illness. Brain Sci..

[66] Tseng P.T., Cheng Y.S., Chen Y.W., Wu C.K., Lin P.Y. (2015). Increased levels of vascular endothelial growth factor in patients with major depressive disorder: A meta-analysis. Eur. Neuropsychopharmacol..

[67] Chen M., Zhang L., Jiang Q. (2020). Peripheral IGF-1 in bipolar disorder and major depressive disorder: A systematic review and meta-analysis. Ann. Palliat. Med..

[68] Fernández-Pereira C., Penedo M.A., Rivera-Baltanás T., Pérez-Márquez T., Alves-Villar M., Fernández-Martínez R., Veiga C., Salgado-Barreira Á., Prieto-González J.M., Ortolano S. (2023). Protein Plasma Levels of the IGF Signalling System Are Altered in Major Depressive Disorder. Int. J. Mol. Sci..

[69] Ozsoy S., Besirli A., Abdulrezzak U., Basturk M. (2014). Serum ghrelin and leptin levels in patients with depression and the effects of treatment. Psychiatry Investig..

[70] Hasler G. (2010). Pathophysiology of depression: Do we have any solid evidence of interest to clinicians?. World Psychiatry.

[71] Marc D.T., Ailts J.W., Campeau D.C., Bull M.J., Olson K.L. (2011). Neurotransmitters excreted in the urine as biomarkers of nervous system activity: Validity and clinical applicability. Neurosci. Biobehav. Rev..

[72] Hughes J.W., Watkins L., Blumenthal J.A., Kuhn C., Sherwood A. (2004). Depression and anxiety symptoms are related to increased 24-hour urinary norepinephrine excretion among healthy middle-aged women. J. Psychosom. Res..

[73] Kaneko F., Kawahara Y., Kishikawa Y., Hanada Y., Yamada M., Kakuma T., Kawahara H., Nishi A. (2016). Long-Term Citalopram Treatment Alters the Stress Responses of the Cortical Dopamine and Noradrenaline Systems: The Role of Cortical 5-HT1A Receptors. Int. J. Neuropsychopharmacol..

[74] Turecki G. (2005). Dissecting the suicide phenotype: The role of impulsive-aggressive behaviours. J. Psychiatry Neurosci..

[75] Müller C.P., Carey R.J., Huston J.P., De Souza Silva M.A. (2007). Serotonin and psychostimulant addiction: Focus on 5-HT1A-receptors. Prog. Neurobiol..

[76] Wisłowska-Stanek A., Kołosowska K., Maciejak P. (2021). Neurobiological Basis of Increased Risk for Suicidal Behaviour. Cells.

[77] Marcinkowska M., Kubacka M., Zagorska A., Jaromin A., Fajkis-Zajaczkowska N., Kolaczkowski M. (2022). Exploring the antiplatelet activity of serotonin 5-HT_2A_ receptor antagonists bearing 6-fluorobenzo[d]isoxazol-3-yl)propyl) motif- as potential therapeutic agents in the prevention of cardiovascular diseases. Biomed. Pharmacother..

[78] Sarawagi A., Soni N.D., Patel A.B. (2021). Glutamate and GABA Homeostasis and Neurometabolism in Major Depressive Disorder. Front. Psychiatry.

[79] Ciranna L. (2006). Serotonin as a modulator of glutamate- and GABA-mediated neurotransmission: Implications in physiological functions and in pathology. Curr. Neuropharmacol..

[80] Duarte N.d.S., Corrêa L.M.d.A., Assunção L.R., de Menezes A.A., de Castro O.B., Teixeira L.F. (2017). Relation between Depression and Hormonal Dysregulation. Open J. Depress..

[81] Feeney J., Kenny R.A. (2022). Hair cortisol as a risk marker for increased depressive symptoms among older adults during the COVID-19 pandemic. Psychoneuroendocrinology.

[82] ajkowska Z., Gullett N., Walsh A., Zonca V., Pedersen G.A., Souza L., Kieling C., Fisher H.L., Kohrt B.A., Mondelli V. (2022). Cortisol and development of depression in adolescence and young adulthood—A systematic review and meta-analysis. Psychoneuroendocrinology.

[83] Fischer S., Strawbridge R., Vives A.H., Cleare A.J. (2017). Cortisol as a predictor of psychological therapy response in depressive disorders: Systematic review and meta-analysis. Br. J. Psychiatry.

[84] Schumacher M.M., Santambrogio J. (2023). Cortisol and the Dexamethasone Suppression Test as a Biomarker for Melancholic Depression: A Narrative Review. J. Pers. Med..

[85] Merali Z., Du L., Hrdina P., Palkovits M., Faludi G., Poulter M.O., Anisman H. (2004). Dysregulation in the suicide brain: mRNA expression of corticotropin-releasing hormone receptors and GABA(A) receptor subunits in frontal cortical brain region. J. Neurosci..

[86] Souza-Teodoro L.H., Davies N.M., Warren H.R., Andrade L.H.S.G., Carvalho L.A. (2024). DHEA and response to antidepressant treatment: A Mendelian Randomization analysis. J. Psychiatr. Res..

[87] Markopoulou K., Papadopoulos A., Juruena M.F., Poon L., Pariante C.M., Cleare A.J. (2009). The ratio of cortisol/DHEA in treatment resistant depression. Psychoneuroendocrinology.

[88] Nuguru S.P., Rachakonda S., Sripathi S., Khan M.I., Patel N., Meda R.T. (2022). Hypothyroidism and Depression: A Narrative Review. Cureus.

[89] Pae C.U., Mandelli L., Han C., Ham B.J., Masand P.S., Patkar A.A., Steffens D.C., De Ronchi D., Serretti A. (2009). Thyroid hormones affect recovery from depression during antidepressant treatment. Psychiatry Clin. Neurosci..

[90] Beurel E., Grieco S.F., Jope R.S. (2015). Glycogen synthase kinase-3 (GSK3): Regulation, actions, and diseases. Pharmacol. Ther..

[91] Duman R.S., Heninger G.R., Nestler E.J. (1997). A molecular and cellular theory of depression. Arch. Gen. Psychiatry.

[92] Hacimusalar Y., Eşel E. (2018). Suggested Biomarkers for Major Depressive Disorder. Noro Psikiyatr. Ars..

[93] Kondo S., El Omri A., Han J., Isoda H. (2015). Antidepressant-like effects of rosmarinic acid through mitogen-activated protein kinase phosphatase-1 and brain-derived neurotrophic factor modulation. J. Funct. Foods.

[94] Chatterjee D., Beaulieu J.M. (2022). Inhibition of glycogen synthase kinase 3 by lithium, a mechanism in search of specificity. Front. Mol. Neurosci..

[95] Kennis M., Gerritsen L., van Dalen M., Williams A., Cuijpers P., Bockting C. (2020). Prospective biomarkers of major depressive disorder: A systematic review and meta-analysis. Mol. Psychiatry.

[96] Dhillon G.K., Gangaram S.C. (2024). Examining potential biomarkers for depression diagnosis: A literature review. Undergrad. Res. Nat. Clin. Sci. Technol..

